# LTBP4 affects renal fibrosis by influencing angiogenesis and altering mitochondrial structure

**DOI:** 10.1038/s41419-021-04214-5

**Published:** 2021-10-13

**Authors:** Chi-Ting Su, Tzu-Ming Jao, Zsolt Urban, Yue-Jhu Huang, Daniel H. W. See, Yao-Chou Tsai, Wei-Chou Lin, Jenq-Wen Huang

**Affiliations:** 1 Renal Division, Department of Internal medicine, National Taiwan University Hospital Yunlin Branch, Douliu, Taiwan; 2 Department of Human Genetics, Graduate School of Public Health, University of Pittsburgh, Pittsburgh, PA USA; 3 Department of Medicine, National Taiwan University Cancer Centre Hospital, Taipei, Taiwan; 4 Department of Medical Education and Research, Kaohsiung Veterans General Hospital, Kaohsiung City, Taiwan; 5 Institute of Precision Medicine, National Sun Yat-sen University, Kaohsiung City, Taiwan; 6 Department of Pathology, National Taiwan University Hospital, Taipei, Taiwan

**Keywords:** Cell signalling, Pathogenesis

## Abstract

Transforming growth factor beta (TGFβ) signalling regulates extracellular matrix accumulation known to be essential for the pathogenesis of renal fibrosis; latent transforming growth factor beta binding protein 4 (LTBP4) is an important regulator of TGFβ activity. To date, the regulation of LTBP4 in renal fibrosis remains unknown. Herein, we report that LTBP4 is upregulated in patients with chronic kidney disease and fibrotic mice kidneys created by unilateral ureteral obstruction (UUO). Mice lacking the short LTBP4 isoform (*Ltbp4*S^*−/−*^) exhibited aggravated tubular interstitial fibrosis (TIF) after UUO, indicating that LTBP4 potentially protects against TIF. Transcriptomic analysis of human proximal tubule cells overexpressing LTBP4 revealed that LTBP4 influences angiogenic pathways; moreover, these cells preserved better mitochondrial respiratory functions and expressed higher vascular endothelial growth factor A (VEGFA) compared to wild-type cells under hypoxia. Results of the tube formation assay revealed that additional LTBP4 in human umbilical vein endothelial cell supernatant stimulates angiogenesis with upregulated vascular endothelial growth factor receptors (VEGFRs). In vivo, aberrant angiogenesis, abnormal mitochondrial morphology and enhanced oxidative stress were observed in *Ltbp4S*^−/−^ mice after UUO. These results reveal novel molecular functions of LTBP4 stimulating angiogenesis and potentially impacting mitochondrial structure and function. Collectively, our findings indicate that LTBP4 protects against disease progression and may be of therapeutic use in renal fibrosis.

## Introduction

Extensive lines of evidence implicate transforming growth factor beta (TGFβ) signalling in lung fibrosis, neoplasm, cardiomyopathy, diabetic nephropathy (DMN), and inflammation, correlating with disease severity in all cases [1]. Thus, understanding the mechanisms of TGFβ activation and TGFβ-related signalling is relevant to developing therapeutic strategies for systemic diseases with major public health impacts. TGFβ acts ubiquitously as a cytokine, playing a crucial role in the synthesis of extracellular matrix (ECM) molecules, contributing to fibrotic disorders and regulating the immune system [2]. In addition, it influences cellular growth, differentiation, and proliferation at various stages of development and maintains tissue homeostasis during adulthood [3]. Latent TGFβ binding proteins (LTBPs) are a group of matrix proteins with important and complicated functions in the ECM. LTBPs directly facilitate TGFβ action through several mechanisms. TGFβ molecules are secreted as dimers, binding via strong non-covalent interactions to their dimeric propeptide, the latency-associated peptide (LAP). The TGFβ-LAP complex represents a small latent complex (SLC), while a tri-partite complex, known as the large latent complex, comprises an LTBP molecule bound to SLC via disulfide bonds [4]. LTBPs mediate targeting of latent TGFβ complexes to ECM structures, a process essential for TGFβ storage and activation, as well as downstream signalling. Thus, LTBPs represent potential regulators of fibrosis in different tissues.

Renal fibrosis is characterized by the abnormal accumulation of ECM, with TGFβ1 as the central mediator, inducing increased matrix protein synthesis and inhibiting matrix degradation [5]. Post-translational regulation of TGFβ1 signalling is a highly relevant aspect of renal fibrosis. LTBPs include four different genes, *LTBP1, LTBP2, LTBP3* and *LTBP4*, where a deficiency of each results in various discrete phenotypes in human [6]. However, as potential regulators of the TGFβ pathway, the role of LTBPs in renal fibrosis remains unclear. One member of the LTBP group, LTBP4, can only bind to TGFβ1, not other TGFβ family members [7], highlighting its potential importance in fibrosis progression.

LTBP4 plays a significant role in elastogenesis and is expressed as two major isoforms, namely long (LTBP4L) and short (LTBP4S) variants synthetized from the same gene by alternate promoter and exon use [8]. Patients with biallelic *LTBP4* mutations have a congenital disorder with a defective elastic fibre network, autosomal recessive cutis laxa type 1C (ARCL1C). The clinical manifestations of ARCL1C are diverse and include craniofacial anomalies, lax skin, and abnormalities in visceral organs, including the lung, heart, kidney, bladder, and gastrointestinal system [9, 10]. Genetic deletion targeting both *Ltbp4* isoforms in mice produces severe neonatal lethality [11], while mice lacking the short *Ltbp4* (*Ltbp4S*^−/−^ mice) replicate the major human phenotype with pulmonary emphysema [11] and, thus, represents a widely accepted animal platform for understanding the pathogenesis of dysregulated LTBP4/TGFβ signalling. Herein, we created a renal fibrosis model in an *Ltbp4S*^−/−^ mouse in an attempt to investigate the mechanisms underlying LTBP4/TGFβ signalling and elucidate the impact of LTBP4 on the pathogenesis of renal tubular interstitial fibrosis (TIF).

## Methods

### Human specimens

Human renal biopsies derived from a normal individual and a patient with chronic kidney disease (CKD) diagnosed with DMN, were purchased from ProteoGenex (Inglewood, Ca, USA). Renal tissue samples were collected from six additional patients with DMN (*n* = 3) or glomerular nephritis (*n* = 3) through our institution and were assessed by immunohistochemistry to detect LTBP4 expression. This study was approved by the Research Ethics Committee C of National Taiwan University Hospital (IRB number is 202101074RINC) and was performed according to the Declaration of Helsinki. Informed consent was obtained from the participants.

### Antibodies

The antibodies used in the study were: rabbit polyclonal antibodies against fibronectin (immunofluorescence staining (IF): 1:100, western blot (WB): 1:1000; Sigma-Aldrich #F3648; St. Louis; USA), collagen I (WB: 1:1000; Thermo Fisher Scientific #PA5-95137; Waltham; USA), cluster of differentiation 31 (CD31, IF: 1:100, WB: 1:1000; Abcam #ab28364; Waltham; USA), 4-hydroxy-2-nonenal (4-HNE; immunohistochemistry (IHC): 1:50; Abcam#46545; Waltham; USA), dynamin-1-like protein (DRP1; WB: 1:1000, Cell Signalling#8570; Danvers; USA) and heat shock protein (HSP60;WB: 1:1000, Cell Signalling#12165; Danvers; USA); mouse monoclonal antibodies against α-SMA-Cy3 (IF: 1:100, WB: 1:1000; Sigma-Aldrich #C6198; St. Louis; USA), vimentin (WB: 1:1000; Santa Cruz Biotechnology #sc6260; Dallas; USA), vascular endothelial growth factor A (VEGFA; WB: 1:1000, IF: 1:100;; Thermo Fisher Scientific #MA5-13182; Waltham; USA), VEGFB (IF: 1:20; Santa Cruz Biotechnology #sc80442; Dallas; USA), mitofusin-2 (MFN2; WB: 1:1000, Abcam#ab56889; Waltham; USA), and glyceraldehyde 3-phosphate dehydrogenase (GAPDH; WB: 1:10,000; Thermo Fisher Scientific #MA5-15738; Waltham; USA); and goat polyclonal antibodies against LTBP4 (WB: 1:800; R&D Systems #AF2885; Minneapolis; USA).

### Cell culture

Cells were obtained from Bioresource Collection and Research Center (BCRC, Hsinchu, Taiwan, ROC). The human proximal tubular cell line HK-2 cultured in Dulbecco’s modified eagle’s medium: nutrient mixture F-12 (DMEM/F12, HyClone; Marlborough; USA) supplemented with 10% foetal bovine serum (FBS; HyClone; Marlborough; USA) at 37 °C in a humidified atmosphere of 5% CO_2_/95% air. Hypoxia was induced by incubating cells for 48 h in a humidified chamber (SCI-tive, Baker Ruskinn; Maine; USA) at 37 °C in an atmosphere of 1% O_2_, 5% CO_2_ and 94% N_2_. Wherever indicated, HK-2 cells were transfected with pcDNA3.1 (Mock) or pcDNA3.1/LTBP4 (LTBP4; OHu23640D, Gene Script; NM_001042545.2; Piscataway; USA) using Lipofectamine 3000 (Invitrogen; Carlsbad; USA) to overexpress LTBP4 protein. The rat kidney fibroblast cell line (NRK-49F) was cultured in DMEM medium (HyClone; Marlborough; USA) supplemented with 5% FBS at 37 °C in a humidified atmosphere of 5% CO_2_/95% air. Human umbilical vein endothelial cells (HUVECs) were cultured in EGM-2 medium (LONZA; Basel; Switzerland) supplemented with 10% FBS in collagen-coated T25 and T75 flasks.

### Co-culture of HK-2 and NRK-49F cells

HK-2 cells were transfected with pcDNA3.1 (Mock) or pcDNA3.1/LTBP4 (LTBP4) using Lipofectamine 3000 (Invitrogen; Carlsbad; USA) to overexpress LTBP4 protein. Two days post-transfection, HK-2 cells were seeded on the Transwell inserts (Corning; NY; USA) and transferred to wells containing NRK-49F cells. On day 4, RNA was harvested from NRK-49F cells and subjected to reverse transcription–quantitative polymerase chain reaction (RT-qPCR).

### Animals and genotyping

*Ltbp4*S−/− mice were a generous gift of Daniel B. Rifkin and were previously described [12, 13]. The genotypes of the offspring from intercrossing heterozygous *Ltbp4S*^+/−^ mice were determined by multiplex PCR. The primer sequences were forward primer 1, 5′-GGCTCATGCTTGAATGTTTCAG-3′; forward primer 2, 5′-ATCATGCAAGCTGGTGGCTG-3′; reverse primer, 5′-CCAATCTTGCTTCTTTGCTGAGC-3′.

### Unilateral ureteral obstruction (UUO) in mice

Animal experiments were conducted following standards and procedures approved by the Ethics Committee for Animal Care and Use of the National Taiwan University College of Medicine. All procedures were carried out in accordance with institutional guidelines for animal experimentation. The C57BL/6 mice and *Ltbp4*S−/− mice (8–9-weeks old; eight to ten per experimental group) were used for establishing the UUO model. The left kidneys of the mice were exposed through a midline incision under sterile conditions; the ureter was then dissected and securely tied at two places with 6.0 silk sutures. To prevent volume depletion, 0.1 ml saline was administrated into the peritoneal cavity. The midline incision was closed, and the mice were returned to their cages with free access to food and water. As a control, sham surgery was performed the same way as UUO without tying the ureter. On the day indicated, the mice were euthanized and the left kidneys from UUO and sham-operated mice were collected for protein extraction, RNA isolation, and histological evaluation.

### RNA extraction and quantitative real-time PCR

Total RNA was extracted from NRK-49F cells, HUVECs, HK-2 cells using RNeasy Mini Kits (Qiagen; Germantown; USA), and 1 μg of total RNA was reverse-transcribed using SuperScript IV Reverse Transcriptase (Invitrogen; Carlsbad; USA), according to the manufacturer’s instructions. The resulting cDNA was used as a PCR template. mRNA levels of genes were determined by quantitative real-time PCR with SYBR Green Real-Time PCR Master Mix (Thermo Fisher Scientific; Waltham; USA) using the CFX Connect Real-Time PCR Detection System (Bio-Rad; Irvine; USA) according to the manufacturer’s instructions. *GAPDH* was used as an internal control. Relative gene expression levels were calculated using the comparative Ct method formula (2^−ΔCt^). Primer sequences are listed in Supplemental Table S1.

### Library preparation and RNA-sequencing

HK-2 cells were transfected with Mock (*n* = 1) or LTBP4 (*n* = 1) using Lipofectamine 3000 (Invitrogen; Carlsbad; USA) to overexpress LTBP4 protein. Two days post-transfection, RNA was extracted from HK-2 cells by the RNeasy Mini Kit (Qiagen; Germantown; USA). All procedures were carried out according to the manufacturer’s protocol for library preparation and RNA-sequencing. Library constructions for all samples were used with Agilent’s SureSelect Strand Specific RNA Library Preparation Kit for 150-bp Paired-End sequencing on a Solexa platform. The sequence was determined using the sequencing-by-synthesis technology with the TruSeq SBS Kit. Raw sequences were obtained from the Illumina Pipeline software bcl2fastq v2.0, and we generated 20 million reads per sample. Gene set enrichment analysis was performed on hallmark pathways to assess the functional enrichment associated with LTBP4 transfectants.

### Tube formation assay in HUVECs

HUVECs (1 × 10^4^) in a 25 μl cell suspension were mixed with 25 μl of growth factor-reduced Matrigel (Corning; NY; USA). Fifty-microliter aliquots were spotted in quadruplicates onto a 24-well plate. After polymerization, spots were covered with designed media samples. Media samples were collected with a protease inhibitor cocktail (Sigma-Aldrich; St. Louis; USA) after treating HEK293T, Mock-transfected HEK293T, and LTBP4-overexpressing HEK293T cells overnight in serum-free media. Amicon Ultra-15 centrifugal filter units (Millipore; Billerica; USA) were used to concentrate media samples. The 1:100 diluted condition media with EGM-2 media were changed every day, and after 48 h, cells were labelled with Calcein AM green dye (Thermo Fisher Scientific; Waltham; USA). Nine images per spot were captured using confocal microscopy (ImageXpress Micro Confocal, Molecular Devices; San Jose; USA). Tubulogenesis was analysed using ImageJ software. Experiments were independently repeated a minimum of three times.

### Measurement of the number of vessels

Microscope fields were observed at a magnification of ×400 and the number of vessels staining positive for CD31 in a single field were counted. The next field was selected randomly by picking the view after four field widths. This process was repeated and vessel counts were obtained for ten random fields. The median value was calculated for each specimen and expressed as count per high-power field (hpf). One observer, blinded to patient details and outcomes, performed the main analysis. A second observer independently counted the number of vessels using the same technique to assess interobserver variability. Furthermore, additional sections from three of the specimens were stained on a different date in order to assess the reliability of the staining methods.

### Measurement of mitochondrial function

Mitochondrial function was measured using a Seahorse flux analyser (Agilent; Santa Clara; USA) according to the procedure provided by the manufacturer. LTBP4 transfectants were seeded in an XF24 cell culture microplate at 1 × 10^4^ cells per well. Mitochondrial function was evaluated by determining OCR and ECAR at baseline and after the addition of oligomycin (1 µM), FCCP (1 µM), and rotenone/antimycin A (0.5 μM). The experiments were repeated independently a minimum of three times with three technical replicates per experiment and biological replicates.

### Western blot analysis

Mouse kidneys and cells were lysed in T-PER (Thermo Fisher Scientific; Waltham; USA) or lysis buffer (25 mM Tris-HCl pH 7.6, 150 mM NaCl, 1% NP-40, 1% sodium deoxycholate, 0.1% SDS), respectively, supplemented with a protease inhibitor cocktail (Thermo Fisher Scientific) on ice for 30 min. Protein concentrations were determined by the BCA Protein assay (Thermo Fisher Scientific; Waltham; USA). Forty micrograms of protein per sample were mixed with Laemmli’s sample buffer and boiled for 10 min. The samples were separated on 10% sodium dodecyl sulfate polyacrylamide gels and transferred to polyvinylidene difluoride membranes (Millipore; Billerica; USA). The blotted membranes were blocked in 5% non-fat milk in TBST (Tris-buffered saline/0.2% Tween 20) for 1 h at room temperature (RT) and incubated with primary antibodies overnight at 4 °C. Membranes were then washed in TBST and incubated with horseradish peroxidase-conjugated secondary antibodies for 1 h at RT. Immunoreactive signals were detected using the chemiluminescent reagent ECL (Millipore #WBLUR0500; Billerica; USA). The experiments were repeated independently a minimum of three times.

### Immunohistochemical staining

Antibody staining was performed on serial 4 μm sections after microwave-mediated antigen retrieval in 10 mM citrate buffer (pH 6.0) by heating for 5 min. Peroxidases were quenched with 3% H_2_O_2_ for 10 min. Slides were blocked in blocking buffer (2.25% glycine, 0.5% saponin, 1% BSA in PBS) for 10 min at RT, followed by incubation with an anti-LTBP4 antibody for 16 h at 4 °C. Detection was performed using the REAL EnVision Detection System (Dako; Jena; Germany) according to the manufacturer’s instructions. Sections were counterstained with Mayer’s haematoxylin (Sigma-Aldrich; St. Louis; USA), dehydrated, and protected by cover slips. Images were captured in digital format using an OLYMPUS DP72 microscope.

### Immunofluorescence staining

Kidney tissues were fixed in 10% formalin neutral-buffered solution (Wako; Osaka; Japan), dehydrated, and embedded in paraffin. Antibody staining was performed on serial 4 μm sections after microwave-mediated antigen retrieval in 10 mM citrate buffer (pH 6.0) by heating for 5 min. Slides were blocked in blocking buffer (2.25% glycine, 0.5% saponin, 1% BSA in PBS) for 10 min at RT, followed by incubation with primary antibodies for 16 h at 4 °C. Detection was performed using appropriate secondary antibodies conjugated with Alexa-488 or Alexa-594 (Invitrogen) for 1 h at RT. Sections were counterstained with 4′,6-diamidino-2-phenylindole (DAPI; 1 μg/ml; Sigma-Aldrich; St. Louis; USA) for 5 min at RT and protected by cover slips. Images were captured in digital format using an OLYMPUS DP72 microscope. For computer-assisted densitometric image analysis, at least ten random fields per section were captured for quantitative assessment of the area positive for staining using the StrataQuest software (TissueGnostics; LA; USA).

### Electron microscopy

Kidney cortex tissue samples were fixed in 2.5% glutaraldehyde and 2.5% paraformaldehyde in phosphate buffer. The proximal tubular cells were photographed and examined under a transmission electron microscope (JEM-1400, JEOL, MA, USA).

### Angiogenesis Arrays

Dot blotting against 55 angiogenesis-related proteins was performed using Proteome Profiler Human Angiogenesis Array Kit (ARY007, R&D Systems; Minneapolis; USA) based on the manufacturer’s instructions (200 µg of cell lysate per sample was loaded onto the membrane).

### Microarray data set

We analysed LTBP4 expression in data deposited in a public repository; it can be accessed through ArrayExpress: E-MTAB-2502.

### Statistical analysis

All data were analysed for their parametric distribution using Shapiro–Wilk test. Student’s *t*-tests were used for between-group analysis, whereas Mann–Whitney *U* tests were used to compare differences between two independent groups when the dependent variable was nonparametrically distributed. A two-sided *P* value < 0.05 was considered significant for all statistical calculations. Data processing was performed with GraphPad Prism version 9.0c for Windows (GraphPad Software Inc.; San Diego; USA).

## Results

### LTBP4 expression is associated with TIF in mice and humans

**Fig. 1:**
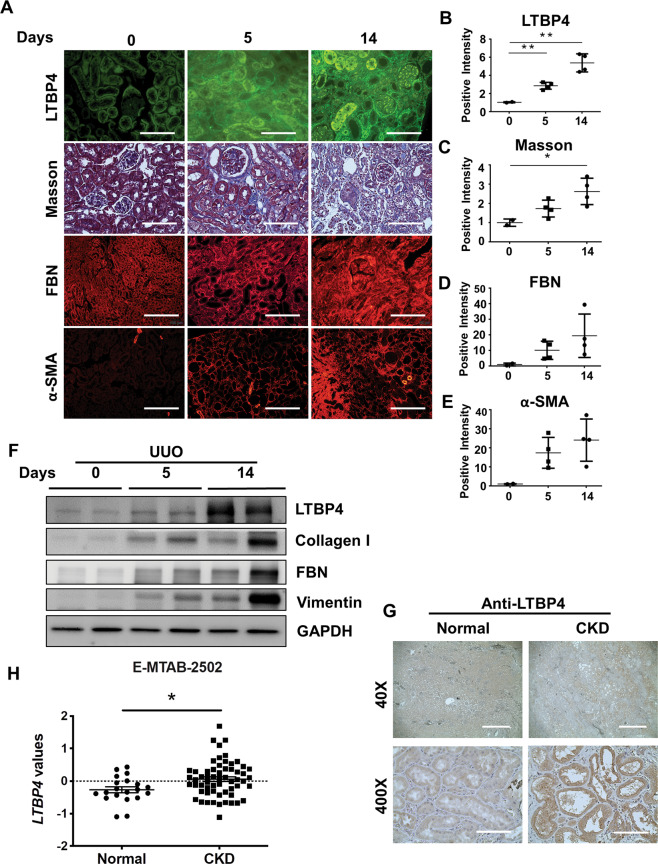
LTBP4 expression is associated with tubulointerstitial fibrosis in mouse and human kidneys. **A**–**F** Wild-type mice were subjected to tubulointerstitial fibrosis by unilateral ureteral obstruction (UUO) for the indicated time points. **A** Representative histological images with Masson’s trichrome and immunofluorescence staining of kidneys after UUO. Scale bars, 100 μm. Computer-assisted quantitative analyses of histological images for Ltbp4 (**B**), Masson’s trichrome (Masson) (**C**), Fibronectin (FBN) (**D**), and α-SMA (**E**) are shown. **F** Protein expression of Ltbp4, collagen I, fibronectin, and vimentin in the kidneys of mice subjected to UUO, as detected by immunoblotting. GAPDH served as an internal control. Representative images from three independent experiments are shown above. *n* = 2 mice in 0-day group; *n* = 4 mice in 5- and 14-day groups. **G** Representative immunohistochemical images showing that LTBP4 was predominantly expressed in the kidneys of a patient with diabetic nephropathy compared to a normal control individual. Scale bars: 1 mm in ×40 and 100 μm in ×400 magnifications. Representative images from three independent experiments are shown above. (**H**) LTBP4 was upregulated in fibrotic kidneys, which was analysed in microarray data set: ArrayExpress_E-MTAB-2502. Normal: health individual; CKD: chronic kidney disease. Data are presented as the mean ± SEM. *p < 0.05, **p < 0.01.

In ongoing efforts to understand the biological functions of LTBP4 in kidney fibrosis, we first measured *Ltbp4* expression in the kidneys of mice subjected to UUO to elucidate the biological roles and molecular mechanisms of *Ltbp4* in TIF. We observed that LTBP4 was upregulated along with TIF progression (Fig. 1A, B, *p* < 0.01) and gradually increasing fibrotic markers, including collagen deposition (Fig. 1A, C, *p* < 0.05), fibronectin (Fig. 1A, D, *p* < 0.05), and alpha-smooth muscle actin (α-SMA; Fig. 1A, E, *p* < 0.05). Immunoblotting analysis identified a positive correlation between LTBP4 and fibrotic markers, including collagen I, fibronectin, and vimentin (Fig. 1F). In human donors with CKD, diagnosed with DMN and glomerular nephritis, LTBP4 was found to be upregulated in kidney tissues compared to that of patients with healthy kidneys (Fig. 1G), indicating that LTBP4 expression was associated with human TIF. Given that LTBP4 was upregulated in fibrotic kidneys, we analysed LTBP4 expression in E-MTAB-2502 dataset form Array Express and consistently, it showed significant upregulation (Fig. 1H, *p* < 0.05).

### Ltbp4 deficiency aggravates TIF in mice with UUO

**Fig. 2:**
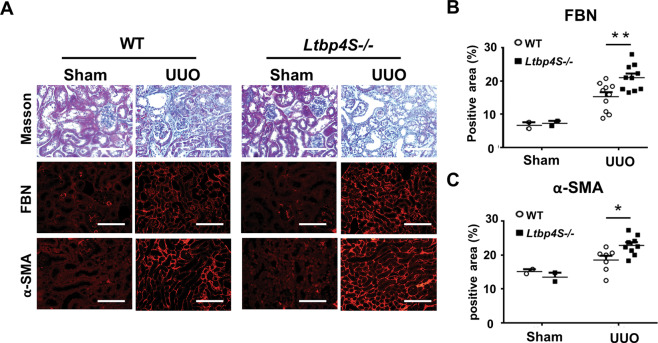
*Ltbp4*S deficiency aggravates tubulointerstitial fibrosis in mice. Wild-type (WT) and *Ltbp4S* knockout (*Ltbp4S*−/−) mice were subjected to unilateral ureteral obstruction (UUO) to induce tubulointerstitial fibrosis for five days. **A** Representative histological images with Masson’s trichrome and immunofluorescence staining of kidneys after UUO. Scale bars, 100 μm. Computer-assisted quantitative analyses of histological images for fibronectin (**B**) and α-SMA (**C**) are shown. Data are presented as the mean ± SEM. *n* = 8–10 mice per group. *p < 0.05, **p < 0.01.

We next investigated the functional consequences of upregulated LTBP4 in fibrotic kidneys and conducted UUO in wild-type (WT) and *Ltbp4S* knockout (*Ltbp4S*^−/−^) mice. *Ltbp4* deficiency led to more severe TIF when compared to WT mice (Fig. 2A–C; Fibronectin: *p* < 0.01; α-SMA: *p* < 0.05), suggesting a protective role for LTBP4 in this fibrotic process.

### Transcriptomic analysis of human proximal tubule (HK-2) cells overexpressing LTBP4

**Fig. 3:**
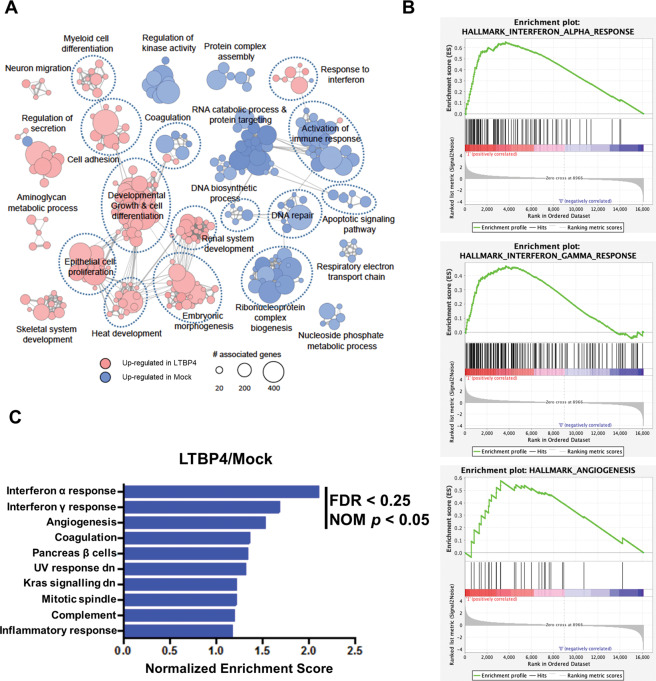
LTBP4 overexpression enriches differentiation, immune response, and angiogenesis phenotypes in HK-2 cells. **A** Enrichment map showing significantly perturbed functions in the LTBP4 overexpression group compared to those in Mock cells based on Gene Ontology gene sets. Edges indicate shared genes. **B** Gene set enrichment analysis (GSEA) plot for representative gene sets enriched in LTBP4 overexpression vs. Mock cells. **C** Top 10 biological functions enriched in LTBP4 overexpression vs. Mock cells. FDR: false discovery rate. NOM *p*: nominal *p*.

To ascertain the molecular mechanisms resulting in LTBP4-medicated renal protection in TIF, we performed transcriptomic analysis of LTBP4-overexpressing or empty vector (Mock) HK-2 cells. Differentially expressed genes between LTBP4-overexpressing and Mock cells were significantly enriched in immune response, cell differentiation, and epithelial cell-associated phenotypes (Fig. 3A), suggesting that LTBP4 could maintain tubular homeostasis and alleviate the mesenchymal phenotypes of fibroblasts. Gene signatures related to interferon response and angiogenesis were enriched in LTBP4-overexpressing HK-2 cells (Fig. 3B, C, Supplemental Table S2). In contrast, gene signatures involved in MYC targets were reduced in HK-2 cells overexpressing LTBP4 (Supplemental Fig. S1 and Supplemental Table S3).

We initially examined whether interferon-γ (IFN-γ) was controlled by LTBP4 to verify the results of the transcriptomic analysis. Upregulated IFN-γ expression was observed in the kidneys of WT and *Ltbp4S*^−/−^ mice subject to UUO when compared to levels in the sham group. However, there were no obvious difference in IFN-γ expression between WT and *Ltbp4S*^−/−^ mice (Fig. S2), suggesting that LTBP4 deficiency did not affect IFN-γ production in renal fibrosis.

Next, we assessed the effect of epithelial LTBP4 on myofibroblast differentiation using an HK-2 cell and NRK-49F rat fibroblast co-culture system (Fig. S3A). Overexpression of LTBP4 in HK-2 cells (Fig. S3B) upregulated the epithelial marker E-cadherin and downregulated the mesenchymal markers fibronectin and collagen I in NRK-49F cells (Fig. S3C). Thus, LTBP4 antagonized the differentiation of fibroblasts into myofibroblasts, a process that plays a major role in renal fibrosis development.

### LTBP4 induces angiogenesis in HUVECs

**Fig. 4:**
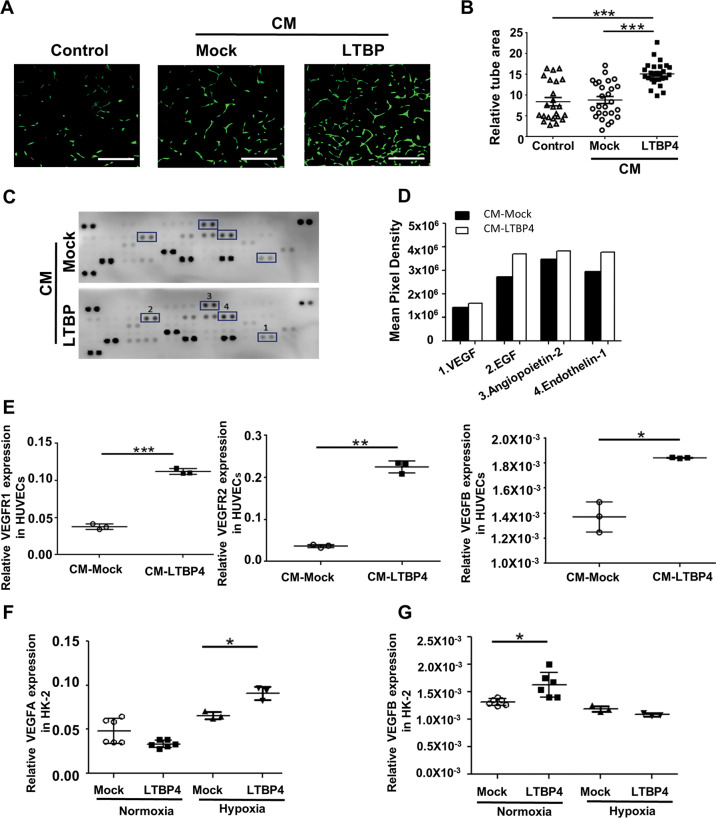
LTBP4 facilitates angiogenesis in HUVECS and HK-2 cells. Conditioned media (CM) collected from Mock (CM-Mock) or LTBP4-overexpressing HEK293T cells (CM-LTBP4) were used to treat human umbilical vein endothelial cells (HUVECs) and human proximal tubule HK-2 cells for 48 h, and tube areas and angiogenic factors were studied. **A** Representative images of tube formation in HUVECs. Control: HUVECs were treated with a regular growth medium. Scale bars, 400 μm. The results are from three independent experiments. **B** Densitometric analyses of tube areas in three groups are shown. **C, D** In proteomic profiling analysis, dot blot profiling for 55 angiogenesis-related proteins showed relatively increasing expression of proangiogenic factors, including VEGF, EGF, angiopoietin-2 and endothelin-1. **E** Relative expression of VEGFRs and VEGF in HUVECs treated with CM-Mock and CM-LTBP4. CM-LTBP4 stimulated VEGFRs and VEGFB expression. **F**, **G** Effect of overexpression of LTBP4 in HK-2 cells. In normoxia, LTBP4 promoted VEGFB expression while, in hypoxia, LTBP4 enhanced VEGFA expression. *p < 0.05, **p < 0.01, ***p < 0.001; VEGF vascular endothelial growth factor, EGF epidermal growth factor, VEGFA vascular endothelial growth factor A, VEGFB vascular endothelial growth factor B, VEGFR1 vascular endothelial growth factor receptor 1, VEGFR2 vascular endothelial growth factor 2.

As LTBP4 was found to exert significant control over angiogenesis in transcriptomic analysis, we next sought to determine whether LTBP4 functionally impacts tubulogenesis in HUVECs, presenting a widely used platform for in vitro studies of the vasculature and angiogenesis. We generated LTBP4-overexpressing HEK293T cells (Fig. S4) and collected supernatants as conditioned media. HUVECs were treated with conditioned media collected from Mock- or LTBP4-overexpressing HEK293T cells (Fig. 4A), after which the areas of tubulogenesis were evaluated. Additional LTBP4 in cell culture media significantly promoted tube formation in HUVECs (Fig. 4B and C; *p* < 0.001). To clarify affected angiogenic molecules in HUVECs, we compared the expression of angiogenesis-related markers between cells treated with and without conditioned media. Proteomic profiling revealed that additional LTBP4 treatment stimulated several angiogenesis-related factors in HUVECs, including vascular endothelial growth factor (VEGF), epidermal growth factor (EGF), angiopoietin-2, and endothelin-1 (Fig. 4D). To understand the molecular impact of LTBP4 on angiogenic signals, we investigated the expression of VEGFs and vascular endothelial growth receptors (VEGFRs) in HUVECs with additional LTBP4 treatment as well as normoxic and hypoxic LTBP4-overexpressing HK-2 cells. Expression of VEGFB (*p* < 0.05) and VEGFR, including VEGFR1 and VEGFR2 (*p* < 0.001 and *p* < 0.01, respectively), were upregulated in the presence of conditioned media with LTBP4 in HUVECs (Fig. 4E). In addition, LTBP4 was found to stimulate VEGFA expression in hypoxic HK-2 cells. (Fig. 4F; *p* < 0.05) but VEGFB expression (Fig. 4G) was not affected significantly.

### Ltbp4 deficiency reduces angiogenesis in injured renal tissues

**Fig. 5:**
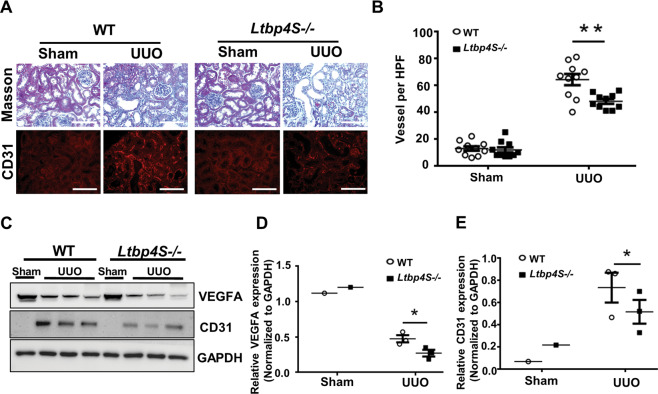
LTBP4 induces angiogenesis for kidney repair in mice subjected to unilateral ureteral obstruction (UUO). Tubulointerstitial fibrosis was induced by UUO for 5 days in wild-type (WT) and *Ltbp4* knockout (*Ltbp4S*−/−) mice. A Representative histological images with Masson’s trichrome and immunofluorescence staining of kidneys for CD31 after UUO. Scale bars, 100 μm. **B** Quantitative results of vessels per HPF in the mouse kidneys. **C** Protein expression of VEGF and CD31 in the kidneys of mice with UUO, as detected by using immunoblotting. GAPDH served as an internal control. **D**, **E** Densitometric analyses of immunoblot images for VEGF (**D**) and CD31 (**E**) are shown. Data are presented as the mean ± SEM for each group (*n* = 3). *P < 0.05, **P < 0.01, ***P < 0.001. Results representative of three independent experiments performed in triplicate.

In injured renal tissues from mice subjected to UUO, on day 5 post-surgery, increased angiogenesis was observed (Fig. 5A, B; *p* < 0.01), however, the response was blunted in *Ltbp4S*^−/−^ mice, suggesting that the increased angiogenesis in mice subjected to UUO was mediated, at least in part, by LTBP4. In injured tissue, angiogenesis was stimulated by various proangiogenic mediators, including VEGFA and transient upregulation of VEGFA expression was detected during renal fibrosis progression (Fig. S5A–B; *p* < 0.05). Furthermore, in injured renal tissues, a higher expression of the angiogenic markers, VEGFA and cluster of differentiation 31 (CD31), was observed in WT mice compared to *Ltbp4S*^−/−^ mice at UUO day 5 (Fig. 5C–E; *p* < 0.05). Additionally, VEGFA and VEGFB expression were reduced in *Ltbp4S*^−/−^ mice at UUO day 5 (Fig. S5C and D).

### LTBP4 preserves the mitochondrial functions in vitro

**Fig. 6:**
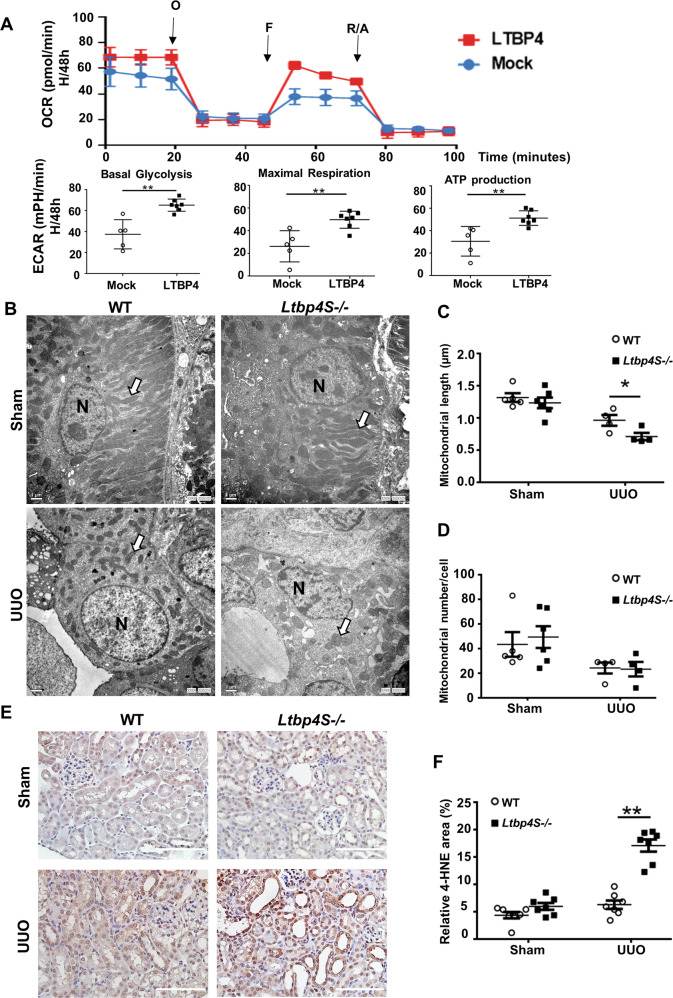
LTBP4 preserves mitochondrial functions in response to hypoxia and Ltbp4 deficiency contributes to fragmented mitochondria and aggravates oxidative stress in mice with unilateral ureteral obstruction (UUO). Seahorse XF Cell Mito Stress Test performed on LTBP4-overexpression human proximal tubule cells cultured under hypoxic conditions (H) for 48 h (H/48 h). **A** OCR profile plot, maximal respiration, ATP production and based glycolysis are higher in hypoxic LTBP4-overexpression cells (LTBP4) compared to hypoxic cells with normal LTBP4 expression (Mock). In vivo, tubulointerstitial fibrosis was induced by UUO for 5 days in wild-type (WT) and *Ltbp4S* knockout (*Ltbp4S*^−/−^) mice. These data were calculated from three independent experiments. Where indicated, O: oligomycin (1 μM), F: FCCP (1 μM) and R/A: rotenone/antimycin A (0.5 μM) were added. Each data point represents the mean ± SD. **B** Representative electron microscopic images of proximal tubular cells of the kidneys of WT and *Ltbp4S*^−/−^ mice with UUO. White arrows indicate mitochondria. N indicates nuclei. Scale bars = 1 μm. Computer-assisted quantitative analyses of electron microscopic images for mitochondrial length (**C**) and number (**D**) are shown. **E**, **F** Assessment of reactive oxygen species by immunohistochemistry staining for 4-HNE in kidney sections. The kidney sections of *Ltbp4S*^−/−^ mice with UUO showed strong positive staining for 4-HNE. Scale Bar = 100 μm. Data are presented as the mean ± SEM for each group of mice (*n* = 4–6 per group). *p < 0.05. 4-HNE: 4-hydroxynonenal. Results are from three independent experiments.

There is compelling evidence that mitochondrial function is associated with angiogenesis [14]. To determine the effect of LTBP4 on mitochondrial functioning upon encountering cellular stress, we compared the mitochondrial bioenergy profile in hypoxic LTBP4-overexpressing and Mock-transfected HK2 cells. Results from Seahorse metabolic measurement showed that oxygen consumption rate (OCR) associated with basal respiration, maximal mitochondrial respiration, and ATP production were significantly enhanced in LTBP4-overexpressing cells (Fig. 6A; *p* < 0.01). Similarly, the extracellular acidification rate (ECAR) associated with basal glycolysis was enriched as well.

### LTBP4 deficiency shortens the mitochondrial length of renal proximal tubular cells in vivo

Herein, we examined whether LTBP4 regulated mitochondrial function in mice TIF. Mitochondrial morphology, length, and number were evaluated in the tubular cells of WT and *Ltbp4S*^−/−^ mice with or without UUO. Interestingly, mitochondrial length (Fig. 6B, C; *p* < 0.05), but not abundance, (Fig. 6B, D) was significantly reduced in *Ltbp4S*^−/−^ mice subjected to UUO when compared to those in WT mice with UUO.

We also determined whether LTBP4 is involved in the pathophysiology associated with changes in mitochondrial fusion and fission. To this end, we studied the protein expression of mitofusin 2 (MFN2), a mitochondrial fusion mediator, and found that its expression tended to be reduced in *Ltbp4S*^−/−^ mice on day 5 after UUO (Fig. S6A, B), and was significantly reduced on day 1 after UUO (Fig. S6D, E and F; *p* < 0.05). This indicated that a decrease in MFN2 exacerbates tubular damage in *Ltbp4S*^−/−^ mice, with subsequent enhancement of mitochondrial fragmentation, which might contribute to kidney fibrosis. Meanwhile, dynamin-related protein 1, a mitochondrial fission mediator, was not significantly different between the WT and *Ltbp4S*^−/−^ mice (Fig. S6A, C). To determine whether altered mitochondrial structures and concurrent mitochondrial dysfunction were developed along with enhanced oxidative stress, we performed IHC staining for 4-hydroxynonenal (4-HNE) and noted its expression after UUO to be significantly higher in *Ltbp4*S^−/−^ mice compared to WT mice (Fig. 6E, F; *p* < 0.01).

## Discussion

We showed that LTBP4 was upregulated in fibrotic kidneys compared to healthy individuals by accessing data in a public repository and in human renal tissue. Using a UUO model in *Ltbp4S*^*−/−*^ mice, we showed that LTBP4 protects against TIF in CKD via non-TGFβ-related signalling, enhancing angiogenesis and altering mitochondrial structure. Reduced angiogenesis, abnormal mitochondrial morphology and enhanced oxidative stress may act in concert to induce more severe fibrosis in *Ltbp4S*^*−/−*^ mice. Here, we also found that LTBP4 could preserve better mitochondrial functioning after hypoxia in vitro. LTBP4 is an essential regulator of the TGFβ pathway, which is known to be strongly associated with fibrosis, indicating that it enhances renal tissue resistance to damage and plays a potentially protective role during CKD progression.

Renal fibrosis is essential for CKD development and progression, and the major mediator is TGFβ1. Several studies have focused on the inhibition of TGFβ1 and its downstream molecules for the treatment of renal fibrosis [15]. TGFβ1 suppresses ECM degradation and stimulates the deposition of ECM, which either directly or indirectly controls TGFβ activation [16]. LTBPs are important regulators of TGFβ activation, and only LTBP4 binds to the TGFβ1 isoform, though inefficiently [13]. LTBP4 can also enhance TGFβ signalling in cells by stabilizing TGFβ receptors on the membrane [17]. We overexpressed LTBP4 in human proximal tubule cells, the major targets of acute and chronic renal injury, and transcriptomic analysis revealed multiple processes associated with altered protein expression, such as angiogenesis and energy usage. The complexity of LTBP4 functions might provide a novel target for the treatment of renal fibrosis.

Reduced angiogenesis, attributed to a lack of LTBP4, has been reported in pulmonary interstitial fibrosis with impaired alveolarization [18]. To date, the current study is the first to show the interplay between LTBP4 and angiogenesis in TIF. Accumulating evidence has demonstrated that LTBP4 is related to non- TGFβ-pathways in many diseases [19]. Herein, additional LTBP4 was observed to induce tubulogenesis in HUVECs by inducing angiogenic factors, including VEGFR1, VEGFR2 and VEGFB; moreover, LTBP4 induced upregulation of VEGFA in hypoxic LTBP4 overexpressing HK-2 cells rather than VEGFB. Conversely, *Ltbp4S*-knockout blunted the expression of CD31, as well as the endothelial markers, VEGFA and VEGFB in mice with UUO. Thus, both gain and loss of function experiments implicated LTBP4 in mediating a protective angiogenic response in TIF. Meanwhile, VEGFB is stable and not influenced by hypoxia or growth factors [20], similar findings were observed in the current study in hypoxic HK-2 (Fig. 4G). Although VEGFB does not play a direct role in angiogenesis under normal conditions or following its overexpression, it has been implicated in the indirect regulation of VEGFA activity [21], and is associated with cell survival [22].

The downregulation of inflammatory gene expression, observed in our transcriptomic studies, may also contribute to the mitigation of TIF by LTBP4. Angiogenesis is known to be an essential component of inflammation and was shown to be significant in the pathogenesis of ischaemic and chronic inflammatory diseases. Moreover, reduced expression of VEGF has been reported in TIF [23–25]. Jonson’s group thoroughly investigated endothelial cell and peritubular capillary loss in renal insufficiency and reported that an increased VEGF expression is transient in the early stage after injury. A subsequently reduced expression of VEGF is associated with peritubular capillary loss, which correlates with the severity of interstitial fibrosis [26, 27]. Herein, we similarly observed transient upregulation of VEGF on day 1 after UUO in WT mice but this phenomenon was lack in *Ltbp4S*^−/−^ mice (Fig. S5A–B). This could be a reason for a greater reduction of VEGF expression in *Ltbp4S*^−/−^ mice than in WT mice on day 5 after UUO. Furthermore, VEGF might influence acute and chronic renal disease-related angiogenesis and its effects could attenuate the disease process [28–30]. In addition, angiogenesis is considered to facilitate the repair of injured tissues, and in diseases, such as glomerulonephritis and tubulointerstitial fibrosis, the delivery of angiogenesis factors and the enhanced intensity of angiogenesis can be therapeutic in accelerating recovery [28, 31]. CD31 is highly expressed on the surfaces of endothelial cells and is a member of the immunoglobulin-superfamily PECAM-1 [32]. We found reduced CD31 expression in *Ltbp4S*-deficient mice with TIF. This finding indicates that decreased numbers of endothelial cells and levels of angiogenic factors might contribute to a more severe fibrosis in *Ltbp4S*^*−/−*^ mice with UUO.

In addition to the impact on the fibrotic expression profile and angiogenesis, we also observed altered mitochondrial morphology in renal proximal tubule epithelial cells. Accumulating evidence indicates that mitochondrial dysfunction and fibrosis are related [33] and some suggest that mitochondrial morphology is associated with organelle functionality, with the potential to change according to different cell conditions [34]. Alterations in mitochondrial transport and distribution are prone to induce bioenergetic impairment; however, in our in vitro study, the overexpression of LTBP4 alone did not induce ATP production nor affect mitochondrial bioenergetics (not shown), while in pre-treated renal proximal tubule cells under hypoxia environment, LTBP4 appeared to preserve more mitochondria functions. Thus, regulation of mitochondrial structure by LTBP4 may be threshold-dependent, such that reduced concentrations of LTBP4 impair mitochondrial structure, while excess LTBP4 does not increase mitochondrial function above normal levels unless cellular stress developed simultaneously. Clarification of the precise role of LTBP4 in mitochondrial structure and function, and the contribution of this mechanism to the pathogenesis of renal fibrosis will require further investigations.

While mitochondrial morphology was fragmented, increased generation of oxidative stress was detected in *Ltbp4S*^−/−^ mice with renal fibrosis compared with WT mice with fibrotic kidneys. Furthermore, in vitro, excess LTBP4 promoted more ATP generation and preserved better mitochondrial respiratory functions after cellular hypoxia. These findings suggested that LTBP4 might play an essential regulatory role during cellular stress and tissue damage (e.g. fibrosis).

Certain limitations were noted in the current study. First, we did not use LTBP4-overexpressing mice to validate the results of in vitro studies. Further, for the in vitro studies, we did not perform experiments involving *LTBP4*-knockdown to strengthen the findings obtained with the *Ltbp4S*^−/−^ mice. Nonetheless, the fibrotic mouse model offered solid evidence of the role played by LTBP4 in TIF, and LTBP4 upregulation was detected in renal tissues from patients with CKD. Thus, we believe that the results are convincing and relevant to TIF and CKD.

**Fig. 7:**
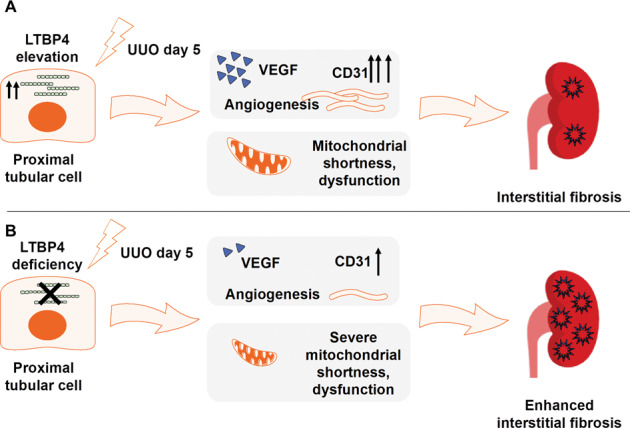
Proposed model of LTBP4-mediated mechanisms in tubular interstitial fibrosis. In unilateral ureteral obstruction (UUO)-induced tubular damage, **A** LTPB4 provokes angiogenesis for tissue repair via VEGF and CD31 elevation and in addition to limiting mitochondrial shortness. **B** LTBP4 deficiency in proximal tubules causes a reduction in VEGF and CD31 expression, along with subsequent aberrant angiogenesis and severe mitochondrial shortness, which eventually accelerates tubular interstitial fibrosis.

In summary, the results presented herein suggest that LTBP4 protects against TIF by enhancing angiogenesis, suppressing inflammatory gene expression, and supporting mitochondrial structure in tubular epithelial cells (Fig. 7). Thus, LTBP4 has multiple, cell-type-specific effects that prevent fibrosis. Biological or pharmacological upregulation of LTBP4 may offer a novel molecular approach to the treatment or management of fibrotic disorders.

## Supplementary information

Supplementary Legends

Supplementary Materials

Reproducibility checklist

## Data Availability

The data that support the findings of this study are available from the corresponding author upon reasonable request.
