# Supplementary material for: LTBP4 affects renal fibrosis by influencing angiogenesis and altering mitochondrial structure
Source: Cell Death Dis. 2021 Oct 13;12(10):943. doi: 10.1038/s41419-021-04214-5 (PMC8514500; doi:10.1038/s41419-021-04214-5)
Supplement: Supplementary file 1 — Supplementary Legends [file 41419_2021_4214_MOESM1_ESM.docx]

**SUPPLEMENTAL MATERIAL**

Supplemental Figure 1. LTBP4 overexpression downregulates MYC signalling in HK-2 cells.

Supplemental Figure 2. Renal interferon-γ expression is detected in wild-type (WT) and *Ltbp4*-knockout (*Ltbp4S*^−/−^) mice subjected to unilateral ureteral obstruction (UUO) for 5 days.

Supplemental Figure 3. LTBP4 counteracts a mesenchymal program in fibroblasts *in vitro*.

Supplemental Figure 4. Overexpression of LTBP4 in HEK293T

Supplemental Figure 5. Expression of VEGF during the progression of tubulointerstitial fibrosis in mice with unilateral ureteral obstruction (UUO).

Supplemental Figure 6. Ltbp4 deficiency alters mitochondrial function in the kidneys of mice with unilateral ureteral obstruction (UUO).

Supplemental Table 1. LTBP4-upregulated signalling pathways analysed by gene set enrichment analysis (GSEA).

Supplemental Table 2. LTBP4-downregulated signalling pathways analysed by gene set enrichment analysis (GSEA).

Supplemental Table 3. Primer sequences used for real-time RT-PCR
