# Supplementary material for: LTBP4 affects renal fibrosis by influencing angiogenesis and altering mitochondrial structure
Source: Cell Death Dis. 2021 Oct 13;12(10):943. doi: 10.1038/s41419-021-04214-5 (PMC8514500; doi:10.1038/s41419-021-04214-5)
Supplement: Supplementary file 2 — Supplementary Materials [file 41419_2021_4214_MOESM2_ESM.docx]

**Supplementary Materials**

**Supplementary Figures**

**A**

**
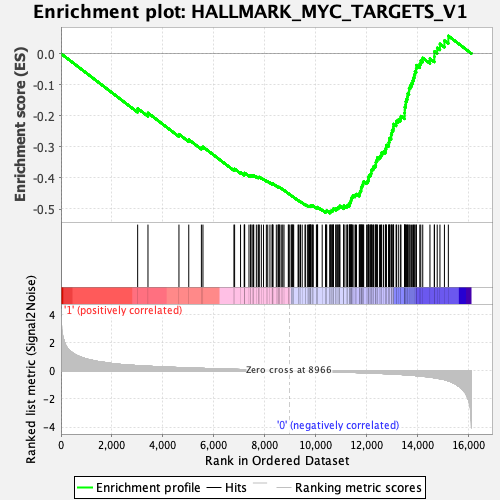

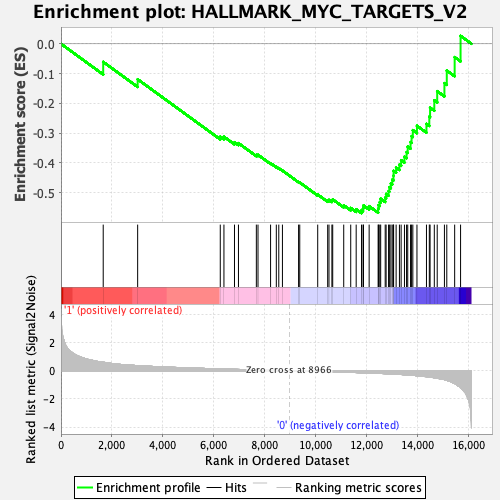
**

**B**

**Fig. S1. LTBP4 overexpression downregulates MYC signalling in HK-2 cells.** (**A**) GSEA plot for representative gene sets reduced in LTBP4-overexpression vs. Mock cells. (**B**) Top 10 biological functions reduced in LTBP4-overexpression vs. Mock cells. FDR: false discovery rate; NOM *p*: nominal; LTBP4: Latent transforming growth factor beta binding protein 4; HK-2 cells: human proximal tubule cells.

**Fig. S2. Renal interferon-γ expression detected in wild-type (WT) and *Ltbp4* knockout (*Ltbp4S-/-*) mice subjected to unilateral ureteral obstruction (UUO) for 5 days.** The interferon-γ expression, observed via immunoblotting, shows no difference between WT and *Ltbp4S-/-* mice. GAPDH served as an internal control. S: Sham.


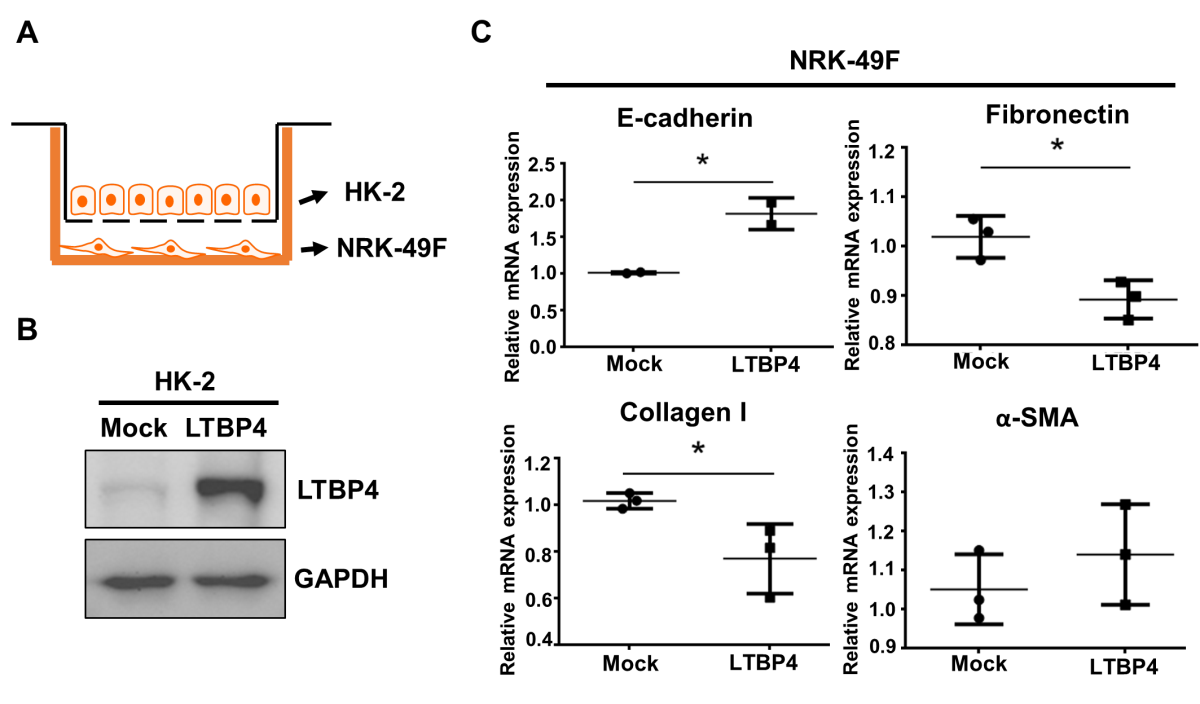


**Fig. S3. LTBP4 counteracts a mesenchymal program in fibroblasts *in vitro*.** LTBP4-overexpressing human proximal tubule HK-2 cells were co-cultured with NRK-49F rat fibroblasts for four days, and epithelial-to-mesenchymal transition (EMT) markers in NRK-49F cells were examined. (**A**) The co-culture system for HK-2 and NRK-49F cells. (**B**) Increased expression of LTBP4 in cell extracts from LTBP4-overexpressing HK-2 cells as confirmed by immunoblotting. (**C**) EMT markers in NRK-49F cells, as measured by qPCR. Data are presented as the mean ± SEM for each group (n = 3). **P* < 0.05.

**Fig. S4.** **Overexpression of LTBP4 in HEK293T.** Supernatants (Sup.) were collected from LTBP4- and Mock-overexpression HEK293T and protein expression of LTBP4 was detected by immunoblotting. FBN served as an internal control. FBN: FIBRONECTIN


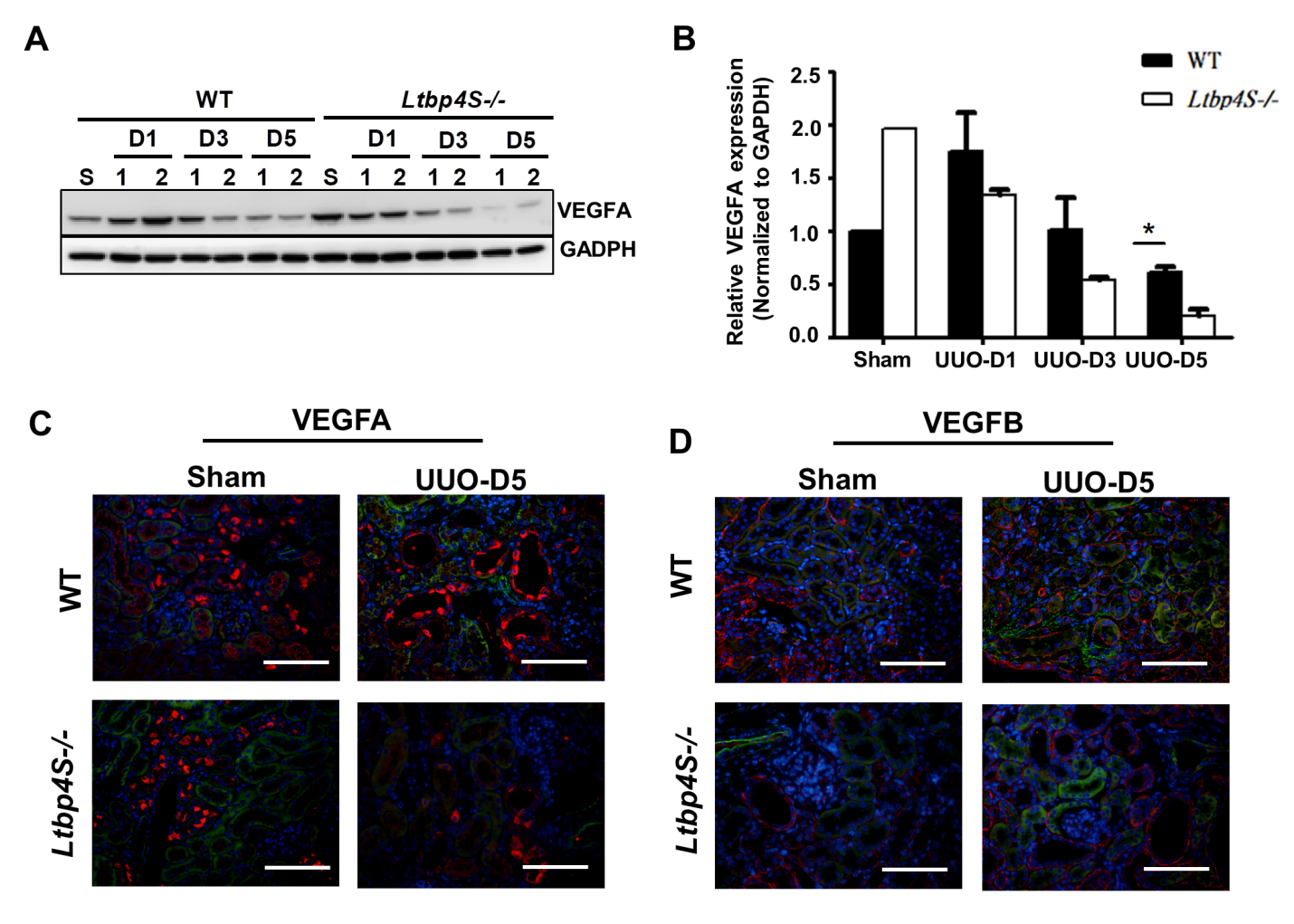


**Fig. S5. Expression of VEGF during the progression of tubulointerstitial fibrosis in mice with unilateral ureteral obstruction (UUO).** (**A**) Protein expression of VEGFA in the kidneys of mice with UUO, as detected by immunoblotting. On day1 after UUO, transient upregulation of VEGFA was detected in WT mice while the stimulation was lack in *Ltbp4*S^-/-^ mice. This may lead to a greater reduction of VEGFA expression in *Ltbp4*S^-/-^ mice on day5 after UUO. GAPDH served as an internal control. S: Sham. D: Day. (**B**) Densitometric analyses of immunoblot images for VEGFA are shown. Data are presented as the mean ± SEM for each group. (C,D) Representative immunofluorescence staining of LTBP4 (green) and VEGF (red). Reduced expression of VEGFA and VEGFB in *Ltpb4*S-/- mice on UUO day 5. Scale bar: 100μm. **p* < 0.05.


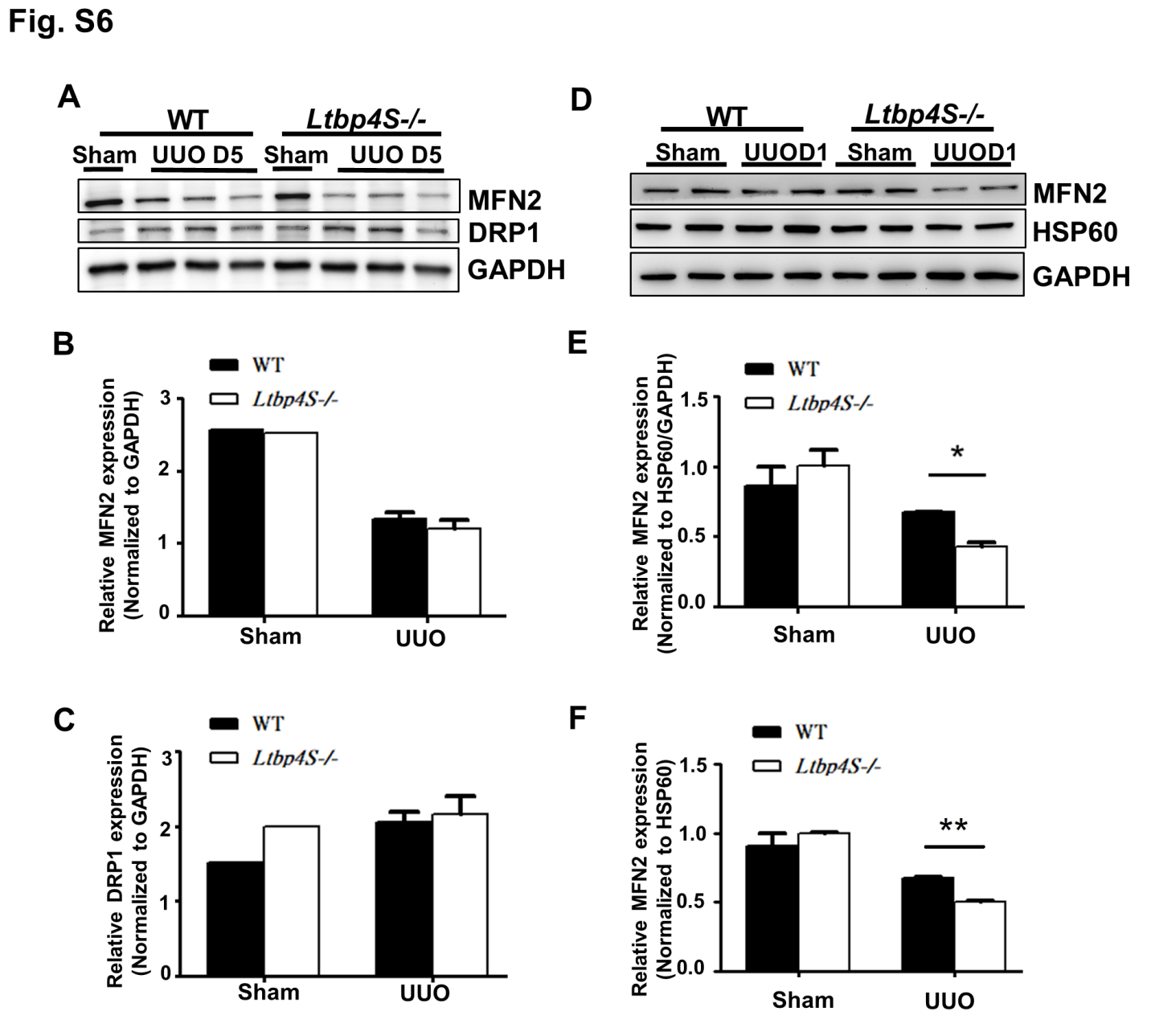


**Fig. S6. Ltbp4 deficiency alters mitochondrial function in the kidneys of mice with unilateral ureteral obstruction (UUO). (A)** On UUO day 5 (UUO D5), protein expression of MFN2 tended to be reduced more whereas DRP1 tended to be increased more in *LTBP4S*-/- mice compared with in wild type (WT) mice. GAPDH served as an internal control. (**B, C**) Densitometric analyses of immunoblot images for MFN2 (B) and DRP1 (**C**) are shown. (**D**) Protein expression of MFN2 and HSP60 in renal tissues from WT mice and *Ltbp4S*-/- mice one day after UUO. MFN2 was significantly reduced more in *Ltbp4S*-/- mice compared in WT mice on UUO day 1 (UUO D1). GADPH served as an internal control. HSP60 is a marker for mitochondrial organelle and was used as a loading control. (**E, F**) Densitometric analyses of immunoblot images are shown. Data are presented as the mean ± SEM for each group. ** *p*< 0.01. MFN2: mitofusin 2; DRP1: dynamin-related protein 1; HSP 60: heat shock protein 60.

**Supplementary Tables**

**Supplemental Table S1. Primer sequences used for real-time RT-PCR**

| **Gene** | **Forward (5ʹ to 3ʹ)** | **Reverse (5ʹ to 3ʹ)** |
| --- | --- | --- |
| *Human* | | |
| VEGFA | AGGGCAGAATCATCACGAAGT | AGGGTCTCGATTGGATGGCA |
| VEGFB | GAGATGTCCCTGGAAGAACACA | GAGTGGGATGGGTGATGTCAG |
| VEGFR1 | TTTGCCTGAAATGGTGAGTAAGG | TGGTTTGCTTGAGCTGTGTTC |
| VEGFR2 | GGCCCAATAATCAGAGTGGCA | CCAGTGTCATTTCCGATCACTTT |
|  | | |
| *Rat* | | |
| E-cadherin | GATCCTGGCCCTCCTGAT | TCTTTGACCACCGTTCTCCT |
| Collagen I | CAGATTGAGAACATCCGCAGC | CGGAACCTTCGCTTCCATACTC |
| Fibronectin | GGGTCACGTACCTCTTCAAAGTCT | CCGTCAGAGGATTGCTTTCC |
| α-SMA | AACTGGTATTGTGCTGGACTCTG | CTCAGCAGTAGTCACGAAGGAATA |
| GAPDH | CAACTCCCTCAAGATTGTCAGCAATG | GGCATGGACTGTGGTCATGA |

**Supplemental Table S2. LTBP4-upregulated signalling pathways analysed by gene set enrichment analysis (GSEA)**

| NAME | SIZE | ES | NES | NOM p-val | FDR q-val | FWER p-val |
| --- | --- | --- | --- | --- | --- | --- |
| HALLMARK_INTERFERON_ALPHA_RESPONSE | 86 | 0.65115803 | 2.1021135 | 0 | 0 | 0 |
| HALLMARK_INTERFERON_GAMMA_RESPONSE | 168 | 0.46845347 | 1.6740323 | 0 | 0.02158437 | 0.04 |
| HALLMARK_ANGIOGENESIS | 27 | 0.5771244 | 1.5270283 | 0.0224 | 0.08470856 | 0.215 |
| HALLMARK_COAGULATION | 94 | 0.41071543 | 1.3525406 | 0.04783951 | 0.35725546 | 0.751 |
| HALLMARK_PANCREAS_BETA_CELLS | 18 | 0.5442873 | 1.33258 | 0.10998308 | 0.34841502 | 0.814 |
| HALLMARK_UV_RESPONSE_DN | 136 | 0.38030797 | 1.3134992 | 0.04689864 | 0.33977017 | 0.862 |
| HALLMARK_KRAS_SIGNALING_DN | 95 | 0.36609638 | 1.2123989 | 0.13629402 | 0.6443727 | 0.992 |
| HALLMARK_MITOTIC_SPINDLE | 197 | 0.33219117 | 1.2086052 | 0.10888252 | 0.5829977 | 0.994 |
| HALLMARK_COMPLEMENT | 159 | 0.3329799 | 1.1890635 | 0.12410842 | 0.5968351 | 0.996 |
| HALLMARK_INFLAMMATORY_RESPONSE | 140 | 0.33276019 | 1.1721619 | 0.15700142 | 0.60068846 | 0.999 |
| HALLMARK_CHOLESTEROL_HOMEOSTASIS | 70 | 0.36897653 | 1.1590987 | 0.21289356 | 0.59611595 | 1 |
| HALLMARK_MYOGENESIS | 145 | 0.3183235 | 1.1215566 | 0.22727273 | 0.69430834 | 1 |
| HALLMARK_EPITHELIAL_MESENCHYMAL_TRANSITION | 186 | 0.3057944 | 1.1140631 | 0.21830986 | 0.6710054 | 1 |
| HALLMARK_BILE_ACID_METABOLISM | 76 | 0.33631498 | 1.0855888 | 0.31124806 | 0.73790586 | 1 |
| HALLMARK_KRAS_SIGNALING_UP | 155 | 0.30078867 | 1.0789713 | 0.28767124 | 0.71473616 | 1 |
| HALLMARK_SPERMATOGENESIS | 78 | 0.32546556 | 1.0528209 | 0.35612082 | 0.77168906 | 1 |
| HALLMARK_ESTROGEN_RESPONSE_EARLY | 179 | 0.2703683 | 0.97111124 | 0.52098405 | 1 | 1 |
| HALLMARK_IL2_STAT5_SIGNALING | 166 | 0.25553516 | 0.91419154 | 0.67902994 | 1 | 1 |
| HALLMARK_APICAL_JUNCTION | 169 | 0.25599304 | 0.9032222 | 0.6896047 | 1 | 1 |
| HALLMARK_XENOBIOTIC_METABOLISM | 146 | 0.2574076 | 0.90116507 | 0.68502825 | 1 | 1 |
| HALLMARK_ESTROGEN_RESPONSE_LATE | 168 | 0.2516697 | 0.8915002 | 0.72988504 | 1 | 1 |
| HALLMARK_REACTIVE_OXYGEN_SPECIES_PATHWAY | 43 | 0.30336466 | 0.8822161 | 0.659396 | 1 | 1 |
| HALLMARK_ADIPOGENESIS | 180 | 0.24052593 | 0.86554927 | 0.80203784 | 1 | 1 |
| HALLMARK_P53_PATHWAY | 183 | 0.23649588 | 0.8572848 | 0.80111265 | 1 | 1 |
| HALLMARK_HEDGEHOG_SIGNALING | 29 | 0.32143962 | 0.8464666 | 0.7035176 | 1 | 1 |
| HALLMARK_FATTY_ACID_METABOLISM | 134 | 0.23805682 | 0.8407147 | 0.81185186 | 1 | 1 |
| HALLMARK_APICAL_SURFACE | 34 | 0.2958782 | 0.8189285 | 0.7296849 | 1 | 1 |
| HALLMARK_ANDROGEN_RESPONSE | 93 | 0.24056852 | 0.7895698 | 0.8840361 | 1 | 1 |
| HALLMARK_IL6_JAK_STAT3_SIGNALING | 64 | 0.24388821 | 0.771622 | 0.85365856 | 1 | 1 |
| HALLMARK_HEME_METABOLISM | 160 | 0.2179255 | 0.7707086 | 0.94842404 | 1 | 1 |
| HALLMARK_APOPTOSIS | 144 | 0.21913166 | 0.76710963 | 0.94126284 | 1 |  |
| HALLMARK_PEROXISOME | 89 | 0.21450856 | 0.6983587 | 0.9691834 | 1 |  |
| HALLMARK_PROTEIN_SECRETION | 90 | 0.20333982 | 0.6721147 | 0.9850075 | 1 |  |
| HALLMARK_HYPOXIA | 175 | 0.17468955 | 0.62601715 | 0.9985316 | 1 |  |
| HALLMARK_PI3K_AKT_MTOR_SIGNALING | 93 | 0.15651648 | 0.51435596 | 1 | 0.99860907 | 1 |

**Supplemental Table S3. LTBP4-downregulated signalling pathways analysed by gene set enrichment analysis (GSEA)**

| NAME | SIZE | ES | NES | NOM p-val | FDR q-val | FWER p-val |
| --- | --- | --- | --- | --- | --- | --- |
| HALLMARK_MYC_TARGETS_V1 | 199 | -0.51331 | 2.0298853 | 0 | 9.38E-04 | 0.001 |
| HALLMARK_MYC_TARGETS_V2 | 57 | -0.5688205 | -1.8446003 | 0 | 0.00186795 | 0.004 |
| HALLMARK_UV_RESPONSE_UP | 142 | -0.311601 | -1.1758685 | 0.13081396 | 0.7483331 | 0.905 |
| HALLMARK_OXIDATIVE_PHOSPHORYLATION | 180 | -0.2588851 | -1.0014864 | 0.42222223 | 1 | 0.999 |
| HALLMARK_ALLOGRAFT_REJECTION | 125 | -0.2660823 | -0.9727198 | 0.49853373 | 1 | 0.999 |
| HALLMARK_TNFA_SIGNALING_VIA_NFKB | 182 | -0.2495286 | -0.9701355 | 0.52459013 | 1 | 0.999 |
| HALLMARK_E2F_TARGETS | 197 | -0.2285845 | -0.8984541 | 0.7682119 | 1 | 1 |
| HALLMARK_NOTCH_SIGNALING | 30 | -0.3133349 | -0.8860273 | 0.64961636 | 1 | 1 |
| HALLMARK_MTORC1_SIGNALING | 196 | -0.205013 | -0.8180345 | 0.9397993 | 1 | 1 |
| HALLMARK_WNT_BETA_CATENIN_SIGNALING | 36 | -0.2435298 | -0.7232368 | 0.8905473 | 1 | 1 |
| HALLMARK_UNFOLDED_PROTEIN_RESPONSE | 109 | -0.1861144 | -0.6728314 | 0.99703264 | 1 | 1 |
| HALLMARK_DNA_REPAIR | 144 | -0.167468 | -0.6418394 | 1 | 1 | 1 |
| HALLMARK_G2M_CHECKPOINT | 194 | -0.1345756 | -0.5299226 | 1 | 1 | 1 |
| HALLMARK_GLYCOLYSIS | 184 | -0.1274736 | -0.5024456 | 1 | 1 | 1 |
| HALLMARK_TGF_BETA_SIGNALING | 53 | -0.1458722 | -0.465533 | 0.9974026 | 0.99938464 | 1 |
